# An immunocompromised patient with cicatricial alopecia

**DOI:** 10.1016/j.jdcr.2025.08.018

**Published:** 2025-08-27

**Authors:** Dominique Revan, Victoria Stoj, Sherry Yang

**Affiliations:** a Jacobs School of Medicine and Biomedical Sciences, University at Buffalo, Buffalo, New York; b Department of Dermatology and Cutaneous Biology, Thomas Jefferson University, Philadelphia, Pennsylvania

**Keywords:** cicatricial alopecia, herpes zoster, varicella-zoster virus

## Case description

**Fig 1:**
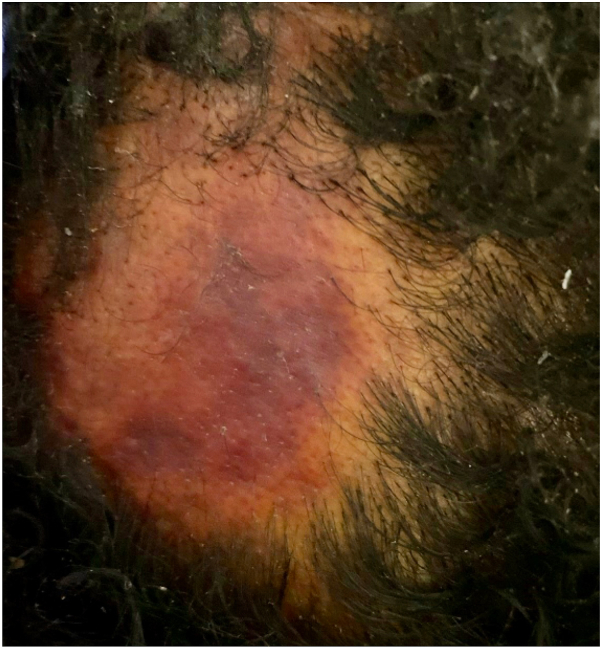
Alopecic patch on the occipital scalp with central erythema and ulceration.

**Fig 2:**
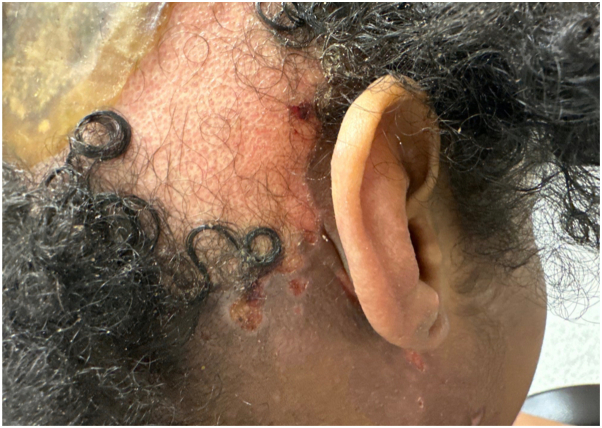
Progression of the alopecic patch on the occipital scalp demonstrating central serous plaque, peripheral punched out erosions, and loss of follicular ostia.

A 37-year-old female with hemophagocytic lymphohistiocytosis presented with sudden onset hair loss that evolved over several hours during admission for neutropenic fever. Examination revealed a well-defined, tender, violaceous alopecic patch with intact follicular ostia (Fig 1). A punch biopsy showed ulcer with nonspecific changes. All fungal, bacterial, and mycobacterial stains were negative. During hospitalization, she was diagnosed with methicillin-resistant staphylococcus aureus (MRSA) bacteremia and discharged on intravenous daptomycin. She presented to the dermatology clinic 1 week later with worsening pain and hair loss, now with loss of follicular ostia (Fig 2).

**Question 1: What is the most likely diagnosis?**

**A.** Varicella-zoster virus (VZV)
**B.** Tinea capitis
**C.** Bacterial abscess due to MRSA
**D.** Discoid lupus erythematosus
**E.** Dissecting cellulitis of the scalp

**Answer Discussion.**

A. Varicella-zoster virus (VZV)—Correct.

Among the answer choices, VZV-associated alopecia is the most likely diagnosis given the rapid onset of painful hair loss in an immunocompromised patient and characteristic punched out erosions visible at the periphery of the alopecic patch in Fig 2. It is hypothesized that VZV-associated alopecia is due to destruction of hair follicles affected by severe inflammatory infiltrate secondary to VZV infection.^1^ Although the initial punch biopsy findings were nonspecific, a diagnosis of VZV-induced alopecia was ultimately confirmed by viral polymerase chain reaction. A small residual patch of scarring alopecia was noted at her 6-month follow-up visit.

Reports of VZV-associated alopecia remain limited in the literature. It is believed that reactivated VZV travels along sensory myelinated nerves, terminating at pilosebaceous units, which may contribute to follicular involvement.^2^ One case series described 2 patients with localized alopecia following VZV infection.^1^ A 4-year-old girl experienced localized hair loss affecting approximately 20% of her upper right eyelash immediately after resolving varicella skin lesions, with no regrowth after 3 months. An 80-year-old woman with a history of alopecia areata (AA) developed a new patch of AA at the site of a vesicular zoster lesion on the left postauricular area. A biopsy was obtained, and she was treated with valacyclovir and clobetasol, leading to complete hair regrowth within 5 months.^1^

The pathophysiologic mechanisms underlying VZV-associated alopecia remain incompletely understood. Active VZV replication within the infundibular keratinocytes of pilosebaceous structures can lead to VZV folliculitis. Hair loss has also been linked to viral infection of peribulbar factor XIIIa-positive (FXIIIa+) dermal dendrocytes, which may initiate a scarring process, ultimately leading to irreversible follicular destruction.^1^ Patients presenting with VZV folliculitis are often immunocompromised due to conditions such as human immunodeficiency virus, hematologic malignancies, or other forms of immunosuppression.^3^ A unique aspect of this case is the patient’s concomitant infection with daptomycin-resistant MRSA, which may have further contributed to her cicatricial alopecia.

In summary, this case of VZV-induced cicatricial alopecia underscores the need for dermatologists to recognize alopecia as a potential manifestation of VZV infection, particularly in immunocompromised patients. Early recognition and treatment may play a role in mitigating permanent follicular damage and improving patient outcomes. Additionally, this case highlights the need for further research into the mechanisms underlying VZV-associated hair loss and potential factors that may contribute to hair regrowth in cases initially presenting as cicatricial alopecia.
